# Role of blister fluid soluble HLA-E In SJS/TEN

**DOI:** 10.1186/2045-7022-4-S3-P48

**Published:** 2014-07-18

**Authors:** Salvador Escamochero, Rosario Cabañas, Ana María Fiandor, Pedro Herranz, Lucia Cachafeiro, Teresa Bellón

**Affiliations:** 1 Hospital La Paz Health Research Institute- IdiPAZ, Research Unit, Spain; 2 Hospital La Paz-IdiPAZ, Allergy, Spain; 3 Hospital La Paz-IdiPAZ, Dermatology, Spain; 4 Hospital La Paz-IdiPAZ, Burn Unit, Spain

## Background

Stevens-Johnson syndrome (SJS) and toxic epidermal necrolysis (TEN) are life-threatening hypersensitivity reactions to medications characterized by keratinocyte apoptosis, the formation of subepidermal blisters, and skin detachment. Cytotoxic lymphocytes including CTLs and NK cells seem to be the main effectors of keratinocyte killing. Natural killer cytotoxic activity is regulated through the balance of activating and inhibitory signals delivered by innate receptors, some of which recognize HLA class I antigens. Among them, inhibitory CD94/NKG2A and activating CD94/NKG2C receptors are specific for the non-classical HLA class Ib molecule HLA-E, and are expressed not only in NK cells but also in subsets of T lymphocytes. We have previously reported that the activating receptor CD94/NKG2C is overexpressed in lymphocytes from SJS/TEN patients resulting in net activation and lysis of HLA-E+ targets. Moreover, HLA-E was found to be overexpressed in affected skin, and in agreement with previous reports showing soluble HLA-E (sHLA-E) released in response to cell activation, we found sHLA-E in blister fluids from SJS/TEN patients.

## Objective

To explore the impact of sHLA-E in the functional capacities of cytotoxic lymphocytes.

## Methods

Conditioned media enriched in sHLA-E or sHLA-B27 (used as a negative control) were prepared and assayed in 51Cr release assays using purified CD94/NKG2A+ or CD94/NKG2C+ primary human NK cells as effector cells. Production of IFN- was also investigated. Western blot was performed to study the phosphorylation status of the signal transducing molecule DAP12.

## Results

The cytotoxic activity and the production of IFN- by NKG2C+ NK cells were significantly increased in the presence of sHLA-E. Moreover, increased phosphorylation of DAP12 was detected. In addition, blister fluids from SJS/TEN patients, were also able to specifically increase the cytotoxic activity of NKG2C+ NK cells, and this effect was reverted in the presence of blocking antibodies against HLA-E.

## Conclusion

sHLA-E acts as an agonist of the activating receptor CD94/NKG2C. The results suggest that sHLA-E molecules in blister fluids may contribute to the massive killing of keratinocytes in SJS/TEN patients by enhancing the activity of NKG2C+ cytotoxic lymphocytes.

